# Heterologous Prime-Boost with ChAdOx1-VZV Establishes Dual-Layer Immunogenicity Conferring Protective Potential Against Herpes Zoster

**DOI:** 10.3390/vaccines13121226

**Published:** 2025-12-05

**Authors:** Jiayu Zhao, Juan Shao, Xiuwen Sui, Menghan Wei, Xinjian Ma, Zhijun Xu, Tao Zhu

**Affiliations:** CanSino Biologics Research Center, Tianjin 300457, China; jiayu.zhao@cansinotech.com (J.Z.); juan.shao@cansinotech.com (J.S.); xiuwen.sui@cansinotech.com (X.S.); menghan.wei@cansinotech.com (M.W.); xinjian.ma@cansinotech.com (X.M.); zhijun.xu@cansinotech.com (Z.X.)

**Keywords:** varicella-zoster virus, ChAdOx1 vectored vaccine, heterologous prime-boost regimen, mucosal immunization

## Abstract

**Background/Objectives**: Varicella-zoster virus (VZV) causes herpes zoster (HZ/shingles), particularly in older adults with weakened cell-mediated immunity (CMI), which is essential for controlling VZV reactivation and reducing HZ severity. Currently vaccines, like recombinant subunit or live-attenuated vaccine, showed shortcomings in eliciting CD8^+^ T-cell responses. Addressing this, we utilized the novel replication-defective chimpanzee adenovirus vector ChAdOx1 to construct the ChAdOx1-VZV (CVE) vaccine, using full-length glycoprotein E (gE) as antigen. This study evaluated the immunogenicity of a heterologous intramuscular (IM) prime/intranasal (IN) boost regimen with the aim of developing a novel VZV vaccine candidate. **Methods**: BALB/c mice were immunized with CVE using homologous or heterologous prime-boost regimens via IM or IN. And cynomolgus macaques were immunized intramuscularly with three doses of CVE. Cellular responses were assessed by intracellular cytokine staining (ICS) and IFN-γ ELISpot using splenocytes and PBMCs. Humoral responses were evaluated by serum gE-IgG ELISA and bone-marrow LLPC ELISpot. Memory subsets and tissue-resident T cells were analyzed by flow cytometry. **Results**: Heterologous IM prime/IN boost CVE regimen markedly enhanced both cellular and humoral responses, especially CD8^+^ T-cell responses. The induced LLPC and memory T cell responses indicate the potential for long-term protection against herpes zoster. In cynomolgus macaques, CVE induced robust serum gE-specific IgG responses and strong IFN-γ secreting T-cell activity, supporting the immunogenicity of CVE in a genetically distinct primate model and enhancing its clinical translational potential. **Conclusions**: CVE induces potent cellular and humoral immunogenicity, with IM prime/IN boost vaccination. Cross species immunogenicity observed in nonhuman primates further strengthens the translational relevance of this platform. These findings support CVE as a promising herpes zoster vaccine candidate and provide a rationale for continued evaluation in human-relevant systems.

## 1. Introduction

Varicella-zoster virus (VZV), a neurotropic alphaherpesvirus, initially manifests as varicella (chickenpox) upon primary infection before establishing lifelong latency in sensory ganglia. Reactivation of the latent virus causes herpes zoster (HZ, shingles), with a global incidence of 10 cases per 1000 person over 60 years old every year [1]. Notably, 5–30% of HZ patients develop postherpetic neuralgia (PHN), and other severe complications include stroke and meningitis [2]. The widely used vaccine Shingrix (GSK), a recombinant subunit vaccine, significantly reduces the risk of herpes zoster by 97.2% in older adults [3]. Although high-efficacy, Shingrix is associated with adverse events [4], including third nerve palsy [5] and retinal necrosis [6]. Meanwhile, live attenuated vaccines face challenges, including age-related waning immunity, residual neurotropism, and contraindications in high-risk groups [7].

VZV-specific cell-mediated immunity (CMI) plays a crucial role in suppressing latent VZV reactivation and promoting HZ recovery. Studies of human ganglia with active HZ or with no history of HZ revealed abundant CD4^+^ T cells and cytotoxic CD8^+^ T cells (Granzyme B^+^) in activated ganglia, with CD8^+^ T cells localized closer to neurons [8]. A cohort study of 12,522 adults aged ≥50 years further demonstrated that VZV-CMI intensity (measured by skin test erythema diameter) correlates with reduced HZ incidence, symptom severity, and PHN risk, whereas humoral responses alone did not correlate with reduced HZ incidence/severity in that cohort [9]. Aging and immunosuppression lead to declined VZV-CMI, increasing HZ recurrence risk [10,11]. These findings highlighted that CD4^+^/CD8^+^ T cells synergistically control the virus during VZV reactivation.

Current vaccines like Shingrix, while effectively eliciting humoral immune responses and CD4^+^ T-cell responses via the AS01B adjuvant system, demonstrate a near inability to induce CD8^+^ T-cell responses [12]. In the design of vaccines aimed at efficiently inducing cellular immune responses, adenovirus vectors are highly favored due to their inherent self-adjuvant effect and potent capacity to induce cellular immunity. Furthermore, chimpanzee adenovirus vectors circumvent prevalent pre-existing immunity challenges due to their low seroprevalence in humans. This advantage is exemplified by the successful deployment of the ChAdOx1 nCoV-19 vaccine (AZD1222, AstraZeneca, Cambridge, UK) against COVID-19. Leveraging this platform, we developed a novel candidate vaccine, ChAdOx1-VZV (CVE), in which the major VZV envelope glycoprotein, full-length of glycoprotein E (gE), serves as the antigen.

Evidence from aerosolized Ad5-nCoV, a replication-deficient human adenovirus type 5 COVID-19 vaccine, has shown that mucosal boosting after prior immunization markedly enhances both systemic and mucosal immunity while requiring lower doses [13,14]. Therefore, we evaluated a sequential regimen consisting of intramuscular (IM) priming followed by intranasal (IN) mucosal boosting in mice. Although animal models do not fully recapitulate VZV latency in humans, this IM-prime/IN-boost (IM + IN) strategy enables assessment of systemic and mucosal cellular responses. We hypothesize that the IM + IN CVE regimen induces a more balanced CD4^+^/CD8^+^ T-cell response, durable antibody production, and enhanced memory formation compared with current subunit vaccination.

## 2. Materials and Methods

### 2.1. Immunization

Female BALB/c mice (6–8 weeks) were immunized intramuscularly (IM) or intranasally (IN) with ChAdOx1-VZV at intervals defined by the study design. Prior to immunization, vaccines were prepared to the appropriate concentration. For IM immunization, mice were injected with 50 µL per dose into the hind limb muscle. For IN immunization, the total volume was 100 µL, administered in two separate doses to avoid expulsion or swallowing. Mice were first anesthetized with isoflurane inhalation and positioned vertically as the nasal cavity, trachea, and lungs were aligned in a straight line. Using a micropipette, approximately 3–5 µL of vaccine suspension was gently applied near one nostril at a time and allowed to be inhaled spontaneously through normal breathing. A total of 50 µL was administered each time, and after approximately 2 h, the same procedure was repeated to deliver the remaining 50 µL.

Saline was used as a negative control, and recombinant herpes zoster subunit vaccine (Shingrix) was used as a positive control. Retro-orbital blood samples were collected, and serum was separated at indicated time points to determine gE-specific antibodies. In addition, spleen, PBMCs, and bone marrow were harvested from experimental mice to evaluate gE-specific T-cell responses and/or antibody-secreting cells, as required by the study design.

Cynomolgus macaque model experiments were conducted by JOINN (Suzhou, China) New Drug Research Center Co., Ltd.

### 2.2. Isolation of Splenocytes, PBMCs, and Bone Marrow Cells

Splenocytes, PBMCs, and bone marrow cells were prepared using standard protocols. For splenocyte isolation, mice were euthanized and surface-sterilized by immersion in 75% ethanol. Spleens were aseptically excised, cut into ~3 mm fragments, and transferred into 2 mL of ACK lysis buffer. Tissues were mechanically dissociated using a DSC-800 tissue grinder (RWD Life Science, Shenzhen, China) for 1 min, followed by red blood cell (RBC) lysis for 4 min at room temperature. The reaction was quenched with 9 mL DMEM containing 1% fetal bovine serum (FBS). Cell suspensions were filtered through a 70 µm strainer, centrifuged, and resuspended in complete RPMI 1640 medium (10% FBS, 1% penicillin–streptomycin).

PBMCs were isolated from peripheral blood collected into anticoagulant-treated tubes. A total of 300 µL of anticoagulated blood was collected form each mouse (*n* = 3~6), with samples from the same group pooled together for easy separation. The blood was then diluted with PBS at a ratio of 5:2. The diluted blood samples were carefully layered onto Ficoll (TBD science, Tianjin, China) in 15 mL centrifuge tubes and centrifuged continuously at 500 g for 30 min at room temperature. The PBMC layer at the plasma–Ficoll interface was collected, washed twice with PBS (250 g, 10 min), and resuspended in complete culture medium.

For bone marrow preparation, femurs and tibias were isolated, immersed in 75% ethanol for 30 s, and rinsed with PBS. Bone ends were cut, and marrow was flushed out with PBS using a syringe. Following centrifugation, the pellet was treated with 1 mL ACK lysis buffer to remove RBCs, and the reaction was stopped with 10 mL RPMI 1640 medium containing 1% FBS. Cells were filtered through a 70 µm strainer, centrifuged, and resuspended in complete culture medium.

All cell preparations were counted, assessed for viability, and used immediately for downstream assays or stored on ice.

### 2.3. ELISA

Purified recombinant gE protein was diluted to 2 μg/mL in sodium carbonate buffer (pH 9.5) and coated onto 96-well plates (100 μL/well) at 4 °C for 18–24 h. Plates were washed with PBST (PBS containing 0.05% Tween-20) and blocked with 1% bovine serum albumin (BSA, Solarbio, Beijing, China) in PBST for 1 h at 37 °C. Serum samples were serially diluted in PBS starting at 1:1000 with a two-fold dilution factor and added to the antigen-coated wells (100 μL/well) for 1 h at 37 °C. After four washes with PBST, HRP-conjugated goat anti-mouse IgG (ZSGB-BIO, Beijing, China) (1:8000 in PBS) was added (100 μL/well) and incubated for 1 h at RT. Plates were developed with TMB substrate according to the manufacturer’s instructions, and the reaction was stopped with 100 μL of 2 M H_2_SO_4_. Absorbance was measured at 450 nm using a microplate reader. The IgG titer was defined as the reciprocal of the highest serum dilution yielding an optical density (OD) ≥2.1-fold above the negative control.

### 2.4. Flow Cytometry

Single-cell suspensions were seeded into 96-well U-bottom plates and stimulated with gE-specific peptide pools (GL Biotech, Shanghai, China). Cells stimulated with PMA and ionomycin (Dakewe, Shenzhen, China) served as positive controls. Cells were incubated at 37 °C in a 5% CO_2_ atmosphere. GolgiStop and GolgiPlug (BD Biosciences, Franklin Lakes, NJ, USA) were added 2 h after stimulation to block cytokine secretion, and incubation continued for another 4 h. Cells were then washed with PBS, stained with Zombie Aqua (BioLegend, San Diego, CA, USA) for live/dead discrimination (RT, 10 min), and subsequently incubated with antibody cocktails for surface markers without washing. Surface staining was performed with CD3-PE-Vio770 (Miltenyi Biotec, Bergisch Gladbach, Germany), CD4-PerCP-Vio700 (Miltenyi Biotec, Bergisch Gladbach, Germany), and CD8-APC-H7 (BD) for another 15 min staining. After PBS washing, cells were fixed and permeabilized overnight at 4 °C using the Fixation/Permeabilization Kit (BD Biosciences, Franklin Lakes, USA), followed by intracellular staining with fluorescent antibodies, and TNFα-FITC (Miltenyi Biotec, Bergisch Gladbach, Germany), IFNγ-PE (Miltenyi Biotec, Bergisch Gladbach, Germany), and IL-2-APC (Miltenyi Biotec, Bergisch Gladbach, Germany) were added and incubated for 30 min at 4 °C.

For memory T-cell surface staining, CD3-PE-Vio770 and CD4-PerCP-Vio700, CD8-APC-H7, CD44-VioBlue (Miltenyi Biotec, Bergisch Gladbach, Germany), CD62L-FITC (Miltenyi Biotec, Bergisch Gladbach, Germany), and CD103-Alexa Fluor (Invitrogen, Carlsbad, USA) were used. And memory T-cell ICS staining was performed using IFNγ-PE and IL-2-APC.

### 2.5. IFNγ-ELISpot

The frequency of gE-specific cytokine-secreting cells was determined by the ELISpot assay. Then, 96-well Multiscreen PVDF plates (Merck Millipore, Burlington, VT, USA) were activated with 50 μL 70% ethanol per well for 1 min, washed five times with distilled water, and coated overnight at 4 °C with anti-mouse IFN-γ coating antibody (Mabtech AB, Nacka, Sweden). After washing, plates were blocked with RPMI-1640 containing 10% FBS (Thermo Fisher Scientific, Waltham, MA, USA) for 2 h at room temperature. Isolated cells (2 × 10^5^ cells/well) were added in the presence of a gE-specific peptide pool (2 μg/mL); wells containing DMSO alone served as background controls. Wells containing 2 × 10^4^ cells stimulated with PMA and ionomycin served as positive controls. Plates were incubated overnight at 37 °C in a 5% CO_2_ atmosphere.

After incubation, plates were washed five times with PBS, and biotin-IFN-γ detection antibody (Mabtech, AB, Nacka, Sweden) was added for 2 h incubation at room temperature. Following five washes, Streptavidin–alkaline phosphatase (ALP) (Mabtech, AB, Nacka, Sweden) was added (100 μL/well) and incubated for 1 h at room temperature. Plates were washed five times, and 100 μL BCIP/NBT substrate (Thermo Scientific, Waltham, USA) was added per well. Spots were developed for 15–45 min at 37 °C, and plates were then thoroughly rinsed with water. Spots were enumerated using a CTL ImmunoSpot S6 Analyzer (ImmunoSpot, Cleveland, OH, USA). Antigen-specific IFN-γ spot counts were calculated after background subtraction (subtracting spots in DMSO wells). Plates were thoroughly rinsed with water, and spot-forming cells (SFCs) were enumerated for duplicate wells using a CTL ImmunoSpot S6 Analyzer. Data are reported as the average SFCs per duplicate well.

### 2.6. B Cell ELISpot

PVDF filter plates were activated by adding 50 μL of 70% ethanol per well for 1 min, followed by five washes with distilled water and coating with 5 μg/well of gE protein overnight at 2–8 °C. Plates were washed five times with PBST and blocked with 5% skim milk for 2 h at RT. Bone marrow cells were plated at 2 × 10^5^ cells/well onto gE-coated membranes and incubated overnight at 37 °C with 5% CO_2_. Plates were then washed five times with PBST, and secreted antibodies were detected by incubation with ALP-conjugated polyclonal goat anti-mouse IgG (100 μL/well; 1:10,000 dilution in PBST) for 2 h at RT. After five washes, 100 μL/well of BCIP/NBT substrate solution was added and allowed to develop for 15–40 min at RT. Plates were thoroughly rinsed with water, and spot-forming cells were enumerated for duplicate wells using a CTL ImmunoSpot S6 Analyzer (ImmunoSpot, Cleveland, OH, USA). Data are reported as the average SFCs per duplicate well.

### 2.7. Statistical Analysis

Data are presented as mean ± SEM for cellular readouts and as geometric mean ± geometric SD for antibody titers, unless otherwise specified. Statistical analyses were performed using GraphPad Prism (version 8). For comparisons involving more than two groups under single or multiple factors, one-way ANOVA or two-way ANOVA followed by Tukey’s multiple-comparisons test was used. When only two groups were compared, unpaired two-tailed t tests were applied. A *p* value < 0.05 was considered statistically significant. *p* < 0.05, *p* < 0.01, *p* < 0.001, and *p* < 0.0001 were indicated as *, **, ***, and ****, respectively.

## 3. Results

### 3.1. ChAdOx1-VZV Characterization

**Figure 1:**
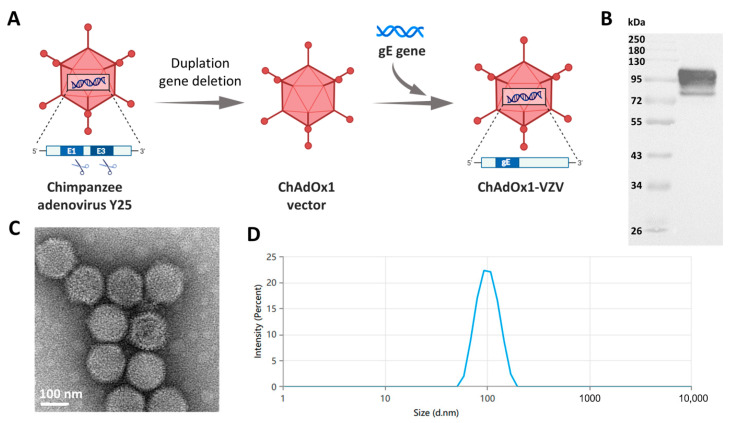
Characterization of ChAdOx1 vector-based VZV vaccine. (**A**) Schematic representation of the construction of ChAdOx1-VZV, generated by deletion of E1/E3 regions and insertion of the codon-optimized VZV gE gene into the ChAdOx1 backbone. (**B**) Western blot analysis confirming in vitro expression of gE glycoprotein in HEK293 cells transduced with ChAdOx1-VZV. (**C**) Transmission electron microscopy (TEM) image showing the typical icosahedral morphology of ChAdOx1-VZV particles. (**D**) Dynamic light scattering (DLS) profile demonstrating a homogeneous particle size distribution.

The zoster vaccine was developed using the ChAdOx1 vector, a replication-deficient chimpanzee adenoviral vector with deletions in the E1 and E3 regions. Full-length glycoprotein E (gE, GenBank accession number AAF61669.1) was inserted into the E1-deleted locus of ChAdOx1 to generate the ChAdOx1-VZV (CVE) vaccine (Figure 1A). gE, a highly conserved and dominant ~98 kDa glycoprotein abundantly expressed on the VZV envelope, exhibits strong immunogenicity by eliciting robust neutralizing antibodies and T-cell responses. Its critical role in viral entry, cell-to-cell spread, and dual glycosylation modifications further establishes it as an optimal vaccine antigen. The unglycosylated gE protein has a molecular weight of approximately 73 kDa. However, in eukaryotic cells, gE undergoes varying degrees of natural glycosylation, leading to a broader molecular weight distribution around 98 kDa. As demonstrated in Figure 1B, gE glycoprotein’s successful expression in HEK293 cells was confirmed by Western blot analysis. The particulate morphology of the adenoviral vector was confirmed by transmission electron microscopy (TEM) (Figure 1C), and dynamic light scattering (DLS) analysis demonstrated a hydrodynamic size distribution consistent with monodisperse adenoviral particles (Figure 1D). Collectively, these results confirm the successful construction of the chimpanzee adenoviral vectored vaccine particle.

### 3.2. Immunogenicity of Single-Dose ChAdOx1-VZV

**Figure 2:**
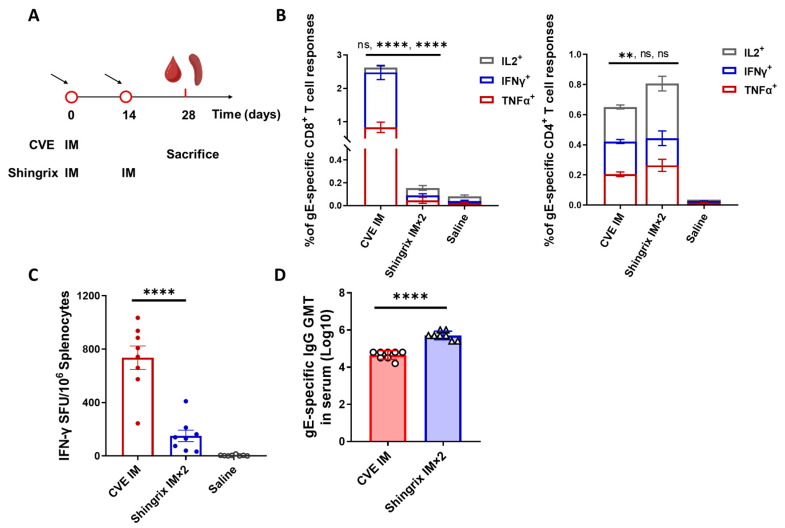
Immunogenicity of single-dose CVE administered intramuscularly (IM) compared with Shingrix. (**A**) Immunization schedule. Female BALB/c mice (6–8 weeks old, 8 mice in each group) received a single IM dose of CVE (5 × 10^9^ viral particles [VPs]/dose) or Shingrix (1/10 human dose, 1/10 HD) at day 0, and Shingrix followed the recommended two-dose regimen with a booster at day 14. (**B**) Intracellular cytokine staining (ICS) analysis of antigen-specific CD8^+^ and CD4^+^ T cells in spleen, showing frequencies of IL-2^+^, IFN-γ^+^, and TNF-α^+^ subsets following stimulation with gE peptide pools (*n* = 8). (**C**) IFN-γ secreting cells in spleen detected by ELISpot assay with gE peptide pool stimulation (*n* = 8). (**D**) Serum gE-IgG geometric mean titers (GMT, log10) measured by ELISA (*n* = 8). Data are shown as mean ± SEM (**B**,**C**) or geometric mean ± geometric SD (**D**). Statistical comparisons by one-way ANOVA or two-way ANOVA in (**B**) and unpaired *t*-test in (**C**,**D**); ns, non-significant; **, *p* < 0.01; ****, *p* < 0.0001.

The immunogenicity of CVE was first evaluated. Naïve female BALB/c mice (6–8 weeks old) were received a single IM dose of CVE (5 × 10^9^ VPs/dose) at day 0. Shingrix controls (one-tenth human dose, 1/10 HD) followed a two-dose schedule (prime on day 0 and boost at day 14) (Figure 2A). Immune responses were analyzed at day 28 (Figure 2B). In intracellular cytokine staining (ICS) assays, IL-2, IFN-γ, and TNF-α cytokines were analyzed as representative markers of T-cell proliferative capacity, cytotoxic/Th1 effector activity, and inflammatory support for antiviral immunity, respectively. The frequencies of single-cytokine positive T-cell subsets are presented as stacked bars in Figure 2B. CVE immunization induced significantly higher CD8^+^ T-cell responses than Shingrix, especially in IFN-γ^+^ and TNF-α^+^ populations. CVE-IM also elicited measurable CD4^+^ T-cell responses, slightly lower than those induced by two-dose Shingrix (Figure 2B). Consistently, IFN-γ Enzyme linked immunospot (ELISpot) assays confirmed that CVE-IM generated significantly more IFN-γ secreting cells than Shingrix, reflecting the strong CD8^+^ T-cell bias of CVE (Figure 2C). As expected, Shingrix produced strong antibody titers after two doses. In contrast, the humoral response in the naïve mouse model after single-dose CVE was modest (Figure 2D). These results suggest that CVE represents a promising vaccine platform for the prevention of herpes zoster, but the imbalance of cellular-humoral responses requires further optimization.

### 3.3. Heterologous Intramuscular–Mucosal Sequential Immunization with CVE

**Figure 3:**
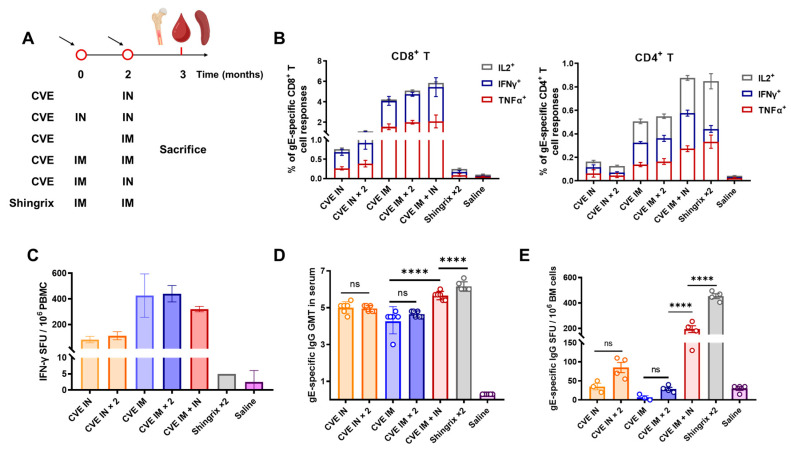
Immunogenicity of CVE under different prime-boost regimens. (**A**) Immunization schedule. Female BALB/c mice (6–8 weeks old, 6 mice in each group) were primed with CVE (IM, 5 × 10^9^ VPs/dose; IN, 2 × 10^9^ VPs/dose) or Shingrix (IM, 1/10 HD) at month 0 and a homologous or heterologous boost (as same dose) at month 2 and were sacrificed one month later. (**B**) ICS of splenic antigen-specific CD8^+^ and CD4^+^ T cells showing frequencies of IL-2^+^, IFN-γ^+^, and TNF-α^+^ subsets after stimulation with gE peptide pools (*n* = 6). (**C**) ELISpot quantification of gE-specific IFN-γ secreting PBMCs (*n* = 2 samples per group, each sample representing PBMCs pooled from 3 mice). (**D**) Serum gE-IgG titers determined by ELISA (*n* = 6). (**E**) Bone marrow gE-specific antibody-secreting cells (ASCs), representing long-lived plasma cells (LLPCs) (*n* = 4, randomly selected from 6 mice in each group). Data are shown as mean ± SEM (**B**,**C**,**E**) or geometric mean ± geometric SD (**D**). Statistical comparisons by one-way ANOVA or two-way ANOVA; ns, non-significant; ****, *p* < 0.0001.

To optimize antibody responses while retaining cellular strength, we compared homologous and heterologous schedules. Six- to eight-week-old female mice were primed on day 0 and boosted at month 2. The single-dose group was immunized on month 2, and humoral and cellular immunity were evaluated one month after the last dose (Figure 3A). And an optimal IN inoculation volume of 100 μL (Figure S1) was firstly determined to ensure efficient pulmonary delivery.

In the comparison of homologous and heterologous vaccination strategies, the IM + IN heterologous regimen demonstrated the most pronounced effects (Figure 3B–E). Spleen T-cell responses were minimal after CVE-IN (single or two-dose). By contrast, following a CVE-IM prime, either an IM or IN boost elicited strong CD4^+^ and CD8^+^ T-cell responses (Figure 3B,C). In particular, the heterologous regimen elevated CD4^+^ T-cell levels to values comparable with Shingrix (Figure 3B). Most importantly, compared to homologous immunization, IM + IN markedly augmented the humoral response, yielding higher serum gE-IgG levels and increased numbers of gE-specific long-lived plasma cells (LLPCs) in the bone marrow (BM) (Figure 3D,E). Although these antibody responses remained lower than those induced by the two-dose Shingrix regimen, CVE compensated for Shingrix’s limited CD8^+^ T-cell induction, thereby addressing this immunological shortcoming. Collectively, these findings indicate that an intranasal CVE boost following intramuscular priming is an effective strategy to elicit robust systemic cellular immunity while simultaneously establishing strong humoral responses.

### 3.4. Memory Responses Induced by Sequential Immunization with CVE

**Figure 4:**
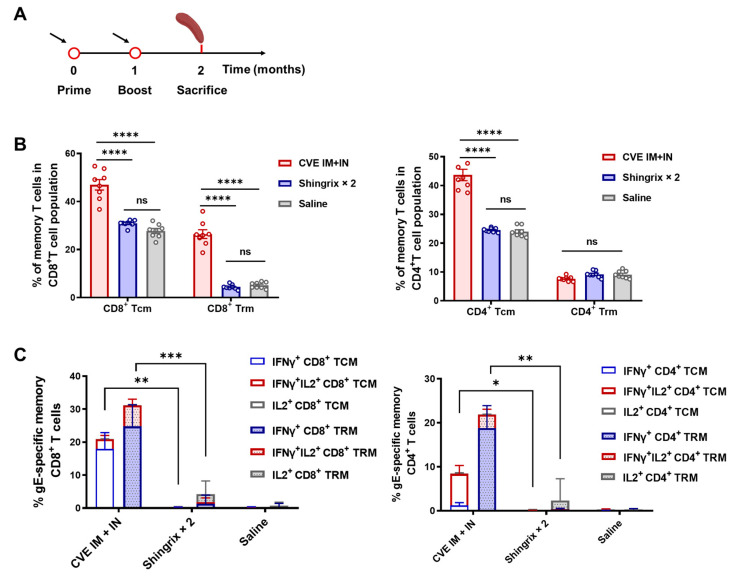
Memory and tissue residency induced by CVE under an IN followed by IM or IM followed by IN heterologous immunization regimen. (**A**) Immunization schedule. Female BALB/c mice (6–8 weeks old, 8 mice in each group) were primed with CVE (IM, 5 × 10^9^ VPs/dose) or Shingrix (IM, 1/10 HD) at month 0, boosted with CVE (IN, 2 × 10^9^ VPs/dose) or Shingrix (IM, 1/10 HD) at month 1, and sacrificed one month later. (**B**) ICS analysis of central memory T cells (TCMs, CD44^+^CD62L^+^) and tissue-resident memory T cells (TRMs, CD44^+^CD62L^−^CD103^+^) within CD8^+^ and CD4^+^ T-cell subsets in spleen (*n* = 8). (**C**) Antigen-specific responses of TCMs and TRMs in spleen, showing frequencies of IFN-γ^+^ and IL-2^+^ memory T cells following stimulation with gE peptide pools (*n* = 8). Data are shown as mean ± SEM. Statistical comparisons by two-way ANOVA; ns, non-significant; *, *p* < 0.05, **, *p* < 0.01, ***, *p* < 0.001, ****, *p* < 0.0001.

Within the sequential immunization framework, intramuscular CVE priming followed by intranasal boosting, we systematically evaluated the quality and magnitude of memory T-cell responses. One month after the final immunization, the frequencies of central memory T cells (TCMs, CD44^+^CD62L^+^) in the spleen were significantly higher in both CD4^+^ T and CD8^+^ T subsets in the CVE group compared with the Shingrix control (Figure 4A,B). The self-renewal capacity and rapid effector differentiation of these TCMs provide a cellular basis for long-term systemic immune surveillance. Furthermore, CVE markedly outperformed Shingrix in inducing CD8^+^ tissue-resident memory T cells (TRMs, CD44^+^CD62L^-^CD103^+^), a population essential for mucosal barrier defense and prevention of herpes zoster reactivation. Antigen-specific assays further confirmed a substantial increase in multifunctional memory T cells (IFN-γ^+^IL-2^+^) in the CVE group (Figure 4C).

Together, these findings suggest that CVE establishes a dual-layer immune memory barrier by combining LLPC-mediated long-term antibody production (Figure 3E) with the expansion of multifunctional memory T-cell subsets, particularly TCMs and TRMs.

### 3.5. Translational Potential Assessment in Non-Human Primate Model

**Figure 5:**
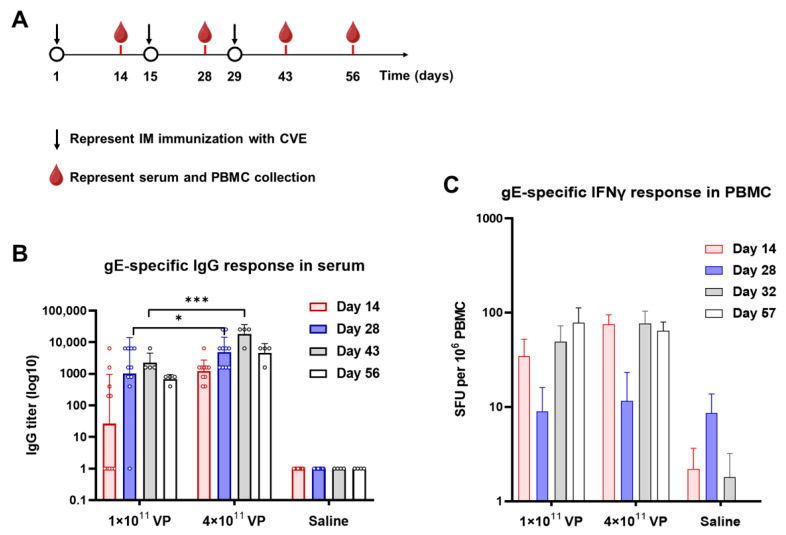
Immunogenicity of CVE vaccine by intramuscular route in cynomolgus macaques. (**A**) Cynomolgus macaques were intramuscularly immunized three doses with ChAdOx1-VZV (CVE) vaccine at 1 × 10^11^ VPs for low dose or 4 × 10^11^ VPs for high dose. Saline-immunized macaques served as a negative control group. There were 10 cynomolgus macaques in each group. (**B**) Serum gE-specific IgG titers at indicated time points post-injection. (**C**) Frequencies of gE-specific IFN-γ secreting cells in the PBMC of experiment monkeys. Data are shown as geometric mean ± geometric SD (**B**) or mean ± SEM (**C**). Statistical comparisons by two-way ANOVA; *, *p* < 0.05, ***, *p* < 0.001.

To further verify translational potential, we also examined the immunogenicity of CVE in a non-human primate (NHP) model using cynomolgus macaques, considering the diversity of MHC haplotypes. And the NHP model could provide a closer approximation to human immune heterogeneity. Following a three-dose IM immunization regimen, CVE elicited robust serum gE-specific IgG responses and strong PBMC IFN-γ secreting T-cell activity in cynomolgus macaques, supporting the broader immunogenic potential of the CVE platform (Figure 5). These studies complement our findings and provide a basis for immunogenicity assessment in other genetic contexts.

## 4. Discussion

This study developed a novel chimpanzee adenoviral vector vaccine, ChAdOx1-VZV (CVE), expressing VZV gE, and showed that a heterologous IM-prime/IN-boost regimen enhances both cellular and humoral responses. CVE induced dominant CD8^+^ T-cell responses and measurable CD4^+^ T-cell responses while establishing LLPCs and memory T cells (TCMs and TRMs), indicating the potential for long-term protection against herpes zoster.

A comparison with current vaccines like Shingrix revealed that CVE effectively elicited humoral immunity and CD4^+^ T cells via AS01B but showed near inability to induce CD8^+^ T-cell responses. Under the heterologous regimen, CVE compensated for this shortcoming by generating robust CD8^+^ T-cell responses while also increasing gE-IgG and LLPCs, though antibody levels remained lower than two-dose Shingrix. This suggests an immunological complementarity in which CVE provides a cellular immune barrier alongside sustained antibody supply. The rationale for the heterologous IM-prime/IN-boost strategy is evidenced from aerosolized Ad5-nCoV clinical trials, which indicate that mucosal boosting after prior immunization can enhance systemic and mucosal immunity at lower doses [13,14]. Consistent with this, our IM + IN regimen elevated CD4^+^/CD8^+^ T cells after IM priming and augmented humoral responses, supporting intranasal boosting as an effective strategy following intramuscular priming.

The induced LLPCs in bone marrow support long-term antibody homeostasis independently of memory B cells [15,16]. The expansion of TCMs provides a basis for systemic immune surveillance, while TRMs contribute to barrier defense. Together, these features suggest a “dual-layer” immune pattern of a cellular immune barrier plus long-term supply of antibodies.

In addition, given that >90% of adults harbor latent VZV with baseline immune memory [17,18,19], a single mucosal dose could provide clinically meaningful protection in populations with widespread pre-existing immunity, particularly for rapid enhancement of cell-mediated immunity or post-exposure scenarios. For individuals with weaker baseline memory, an IM-prime/IN-boost schedule may offer a more balanced and durable enhancement of both antibody and T-cell compartments. Although animal models cannot fully recapitulate human latency, these data provide a rationale for advancing CVE heterologous strategies.

Although murine models are invaluable for evaluating vaccine immunogenicity, they cannot fully reflect the complex biology of VZV latency and reactivation in humans. Particularly, mice cannot mimic VZV latency in humans, which limits the direct assessment of protective efficacy against herpes zoster. This limitation is especially relevant for older adults and immunocompromised individuals, in whom immune activity and impaired CMI elevate the risk of reactivation. Considering that VZV vaccines are primarily intended for older populations, clinical data from Ad5-nCoV vaccination in individuals aged ≥60 years provide relevant translational insight [20]. In elderly participants, Ad5-nCoV elicited moderately reduced antibody responses relative to younger adults, whereas the inactivated vaccine showed a pronounced decline in immunogenicity in older populations. These findings suggest that age-related immune attenuation may differentially influence vaccine platforms, offering a meaningful reference for the development of a ChAdOx1-vectored VZV vaccine.

Moreover, given the restricted susceptibility of murine and other animal models to VZV infection, evaluating true vaccine efficacy remains a major barrier. Human-derived organoid systems therefore represent a promising surrogate platform for future studies on VZV infection biology and antiviral immune responses.

For follow-up clinical research, safety considerations are essential for intranasal adenoviral vaccination. In our preclinical program, we conducted both acute and repeated-dose safety evaluations in SD rats and cynomolgus macaques following IM or IN administration. During the acute safety observation period, no mortality, clinical abnormalities, pathological findings, or adverse effects on body weight or food intake were observed in any animals. In the repeated-dose study, even at high doses up to 4 × 10^11^ VPs/animal administered intramuscularly to cynomolgus macaques, no systemic toxicity was detected. Only transient and reversible increases in body temperature or C-reactive protein were observed, which were consistent with normal vaccine-induced immune activation. Although detailed toxicology datasets are not included here, the available evidence indicates that ChAdOx1-VZV exhibits a favorable acute safety profile in animal models, supporting the feasibility of further development of both intranasal and intramuscular regimens.

## 5. Conclusions

ChAdOx1-VZV (CVE), encoding VZV gE, establishes long-term immunity, including LLPCs and memory T cells (TCMs and TRMs), under a heterologous IM-prime/IN-boost regimen. Together, these features offer a “dual-layer” immune architecture comprising a cellular immune barrier coupled with sustained antibody provision. Although murine models cannot fully model human VZV latency, the immune profile elicited by CVE supports its potential as a candidate herpes zoster vaccine. Together with supportive preclinical safety data in rats and cynomolgus macaques, these findings provide a foundation for advancing CVE development and for future evaluation in human-relevant efficacy systems.

## Data Availability

The datasets used and/or analyzed during the current study are available from the corresponding author upon reasonable request.

## Supplementary Materials

The following supporting information can be downloaded at https://www.mdpi.com/article/10.3390/vaccines13121226/s1, Figure S1: BALB/c mice were intranasally inoculated with a fixed dose of 1 × 10^9^ VP per administration in volumes of 10, 20, 50, or 100 µL. Four hours after inoculation, lungs were harvested, homogenized, and DNA was extracted for quantitative PCR (qPCR) analysis.

## Abbreviations

Ad	Adenovirus
APC	Antigen-presenting cell
BM	Bone marrow
CMI	Cell-mediated immunity
CVE	ChAdOx1-VZV vaccine
DLS	Dynamic light scattering
ELISpot	Enzyme-linked immunospot
gE	Glycoprotein E
HZ	Herpes zoster
IFN-γ	Interferon-gamma
IL-2	Interleukin-2
IM	Intramuscular
IN	Intranasal
ICS	Intracellular cytokine staining
LLPC	Long-lived plasma cell
PHN	Postherpetic neuralgia
TEM	Transmission electron microscopy
TCM	Central memory T cell
TRM	Tissue-resident memory T cell
TNF-α	Tumor necrosis factor-alpha
VZV	Varicella-zoster virus
VP	Viral particle
