# Supplementary material for: Heterologous Prime-Boost with ChAdOx1-VZV Establishes Dual-Layer Immunogenicity Conferring Protective Potential Against Herpes Zoster
Source: Vaccines (Basel). 2025 Dec 5;13(12):1226. doi: 10.3390/vaccines13121226 (PMC12737615; doi:10.3390/vaccines13121226)

Article

Heterologous Prime-Boost with ChAdOx1-VZV Establishes Dual-Layer Immunogenicity Conferring Protective Potential Against Herpes Zoster

Jiayu Zhao, Juan Shao, Xiuwen Sui, Menghan Wei, Xinjian Ma, Zhijun Xu and Tao Zhu *

Supplementary Materials:

Figure S1: BALB/c mice were intranasally inoculated with a fixed dose of 1×10^9 VP per administration in volumes of 10, 20, 50, or 100 μL. Four hours after inoculation, lungs were harvested, homogenized, and DNA was extracted for quantitative PCR (qPCR) analysis.

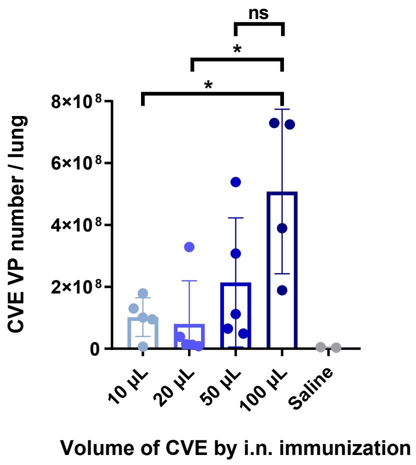

Supplement: Supplementary file 1 [file vaccines-13-01226-s001.zip › vaccines-3989263-supplementary.pdf]
